# Pharmacologic inhibition by spironolactone attenuates experimental abdominal aortic aneurysms

**DOI:** 10.3389/fcvm.2023.1101389

**Published:** 2023-01-26

**Authors:** Zachary Ladd, Gang Su, Joseph Hartman, Guanyi Lu, Sara Hensley, Gilbert R. Upchurch, Ashish K. Sharma

**Affiliations:** Department of Surgery, University of Florida, Gainesville, FL, United States

**Keywords:** abdominal aortic aneurysms, endothelial cells, spironolactone, pannexin 1, macrophages, smooth muscle cells (SMCs)

## Abstract

**Background:**

Abdominal aortic aneurysms (AAA) are characterized by vascular inflammation and remodeling that can lead to aortic rupture resulting in significant mortality. Pannexin-1 channels on endothelial cells (ECs) can modulate ATP secretion to regulate the pathogenesis of AAA formation. Our hypothesis focused on potential of spironolactone to inhibit EC-mediated ATP release for the mitigation of AAA formation.

**Methods:**

A topical elastase AAA model was used initially in C57BL/6 (wild-type; WT) male mice. Mice were administered either a vehicle control (saline) or spironolactone and analyzed on day 14. In a second chronic AAA model, mice were subjected to elastase and β-aminopropionitrile (BAPN) treatment with/without administration of spironolactone to pre-formed aneurysms starting on day 14 and analyzed on day 28. Aortic diameter was evaluated by video micrometry and aortic tissue was analyzed for cytokine expression and histology. ATP measurement and matrix metalloproteinase (MMP2) activity was evaluated in aortic tissue on days 14 or -28. *In vitro* studies were performed to evaluate the crosstalk between aortic ECs with macrophages or smooth muscle cells.

**Results:**

In the elastase AAA model, spironolactone treatment displayed a significant decrease in aortic diameter compared to elastase-treated controls on day 14. A significant increase in smooth muscle α-actin expression as well as decrease in elastic fiber disruption and immune cell (macrophages and neutrophils) infiltration was observed in mice treated with spironolactone compared to saline-treated controls. Spironolactone treatment also significantly mitigated pro-inflammatory cytokine expression, MMP2 activity and ATP content in aortic tissue compared to controls. Moreover, in the chronic AAA model, spironolactone treatment of pre-formed aneurysms significantly attenuated vascular inflammation and remodeling to attenuate the progression of AAAs compared to controls. Mechanistically, *in vitro* data demonstrated that spironolactone treatment attenuates extracellular ATP release from endothelial cells to mitigate macrophage activation (IL-1β and HMGB1 expression) and smooth muscle cell-dependent vascular remodeling (MMP2 activity).

**Conclusion:**

These results demonstrate that spironolactone can mitigate aortic inflammation and remodeling to attenuate AAA formation as well as decrease growth of pre-formed aneurysms *via* inhibition of EC-dependent ATP release. Therefore, this study implicates a therapeutic application of spironolactone in the treatment of AAAs.

## Introduction

Abdominal aortic aneurysms (AAAs) are a leading cause of mortality, especially among the elderly population in the US, with no specific medical therapies available (1–4). The hallmarks of AAAs involve infiltration of immune cells, like macrophages and neutrophils, into the aortic wall with subsequent destruction of elastin and collagen in the media and adventitia by increase in pro-inflammatory cytokine milieu and matrix degrading enzymes such as matrix metalloproteinase (MMP)-2 and -9 (5–10). This leads to thinning of aortic wall and smooth muscle cell (SMC) release of matrix degrading enzymes, such as matrix metalloproteinase 2 (MMP2), which mediates aortic aneurysm pathogenesis (11, 12). The role of aortic endothelial cells (ECs) and its crosstalk with immune cells (macrophages) and SMCs was recently described as we demonstrated that EC-dependent pannexin-1 (Panx 1) channels supports the flux of signaling mediators like ATP that modulate aortic inflammation and vascular remodeling *via* activation of purinergic signaling resulting in AAA formation (13).

Pannexin 1 (Panx 1) is ubiquitously expressed in many tissue and various cells including ECs and SMCs of the vasculature (14, 15). Pannexin channels have been described in humans (PANX1-3), and several disease processes such as cell death, metastasis, leukocyte migration, and neuronal survival have been linked to Panx 1 regulation (16–20). Panx 1 activation can occur *via* caspase-dependent cleavage or receptor-mediated activation that is cleavage-independent (21, 22). Under pathological conditions, Panx 1 can oligomerize to form heptameric channels to act as a conduit for ATP release (21, 22). The accumulation of ATP intracellularly followed by release of extracellular ATP (eATP) acts as a danger associated molecular pattern (DAMP) molecule which initiates tissue inflammation under pathological conditions.

A recent study screened various small molecules targeting Panx 1 and identified spironolactone as a potent inhibitor of Panx 1 channels (23). In this study, the effect of spironolactone on Panx 1 currents induced by α1-adrenergic receptor activation inhibited both mouse and human PANX 1 channels. A key metabolite regulated by Panx 1 channels is ATP that can exert biological actions *via* purinergic P2X and P2Y receptors (16, 22, 24, 25). Macrophage infiltration and activation during AAA is a key immunological event and has been shown to mediate tissue inflammation *via* inflammasome activation (IL-1β) and HMGB1 release during the progression of AAAs (26, 27). The communication between Panx 1 channels on ECs to modulate purinergic signaling on macrophages and SMCs can lead to an inflammatory cascade stimulating the vascular remodeling observed in AAAs (13). Thus, effective pharmacological modalities are required to inhibit this signaling cascade to protect against vascular inflammation.

In this study, using two distinct experimental murine AAA models, we documented that pharmacological inhibition *via* spironolactone causes mitigation of vascular inflammation and remodeling during the pathogenesis of AAA. The mechanistic signaling of eATP release from ECs regulates macrophage activation, which was blocked by spironolactone treatment of ECs. Moreover, eATP release from ECs also activated SMCs resulting in an increased MMP2 activity leading to AAA formation, which was inhibited by treatment of ECs with spironolactone. Blockade by spironolactone leads to significant downregulation of leukocyte infiltration and protection of aortic integrity during experimental AAA formation. Collectively, these data demonstrate that inhibition of aortic endothelial cell-dependent ATP release by pharmacological treatment with spironolactone attenuates AAA formation, and could potentially protect against aortic rupture.

## Materials and methods

### Study design and approval

Animal experiments in this study were conducted under animal protocol approved by the University of Florida’s Institutional Animal Care and Use Committee.

### Animals and reagents

8–12-week-old male C57BL/6 WT mice (Jackson Laboratory, Bar Harbor, ME) were used for this study. A topical elastase treatment murine model of AAA formation was used and phenotype was evaluated on day 14, as previously described (28). The abdominal aorta was treated topically with 30 μl of type 1 porcine pancreatic elastase (5 U/mg of protein) on day 0, with/without administration of Panx1 inhibitor, spironolactone (1.5, 5, or 50 mg/kg intraperitoneally) given from days 1 to 13. In a second model of chronic AAA which is associated with thrombus formation and aortic rupture, mice were treated with topical elastase and 0.2% β-aminopropionitrile (BAPN) with/without spironolactone treatment and analyzed on day 28. The change in aortic diameter was quantified by video micrometry on days 14 or 28 and expressed as percentage increase over baseline aortic diameter. Aortic phenotype were measured by video micrometry using NIS-Elements D5.10.01 software attached to the microscope (Nikon SMZ-25; Nikon Instruments, Melville, NY). Percentage increase in aortic diameter was determined by [(maximal AAA diameter–self-control aortic diameter)/(self-control aortic diameter)] × 100, and aortic dilation of ≥100% was considered positive for AAA formation.

### Histology

Murine abdominal aortas were harvested on days 14 or 28 for immunostaining by histochemistry, as previously reported (29). Antibodies used for histochemical staining were anti-mouse α smooth muscle actin (α-SMA; 1:1000; Sigma, St. Louis, MO), anti-mouse Mac2 for macrophages (1:10,000; Cedarlane Laboratories, Burlington, ON, Canada), and anti-mouse neutrophils for polymorphonuclear neutrophils (PMNs) (1:10,000; AbD Serotec, Oxford, United Kingdom). Aortic sections were also stained with Verhoeff-Van Gieson (VVG) for evaluation of elastin (Polysciences, Inc., Warrington, PA). Images were acquired using AxioCam Software version 4.6 and an AxioCam MRc camera (Carl Zeiss Inc., Thornwood, New York). Threshold gated positive signal was detected within the AOI and quantified using Image-Pro Plus version 7.0 (Media Cybernetics Inc., Bethesda, MD).

### ATP quantification

Quantification of ATP in cell culture supernatants or aortic tissue was performed by a luciferase-based ATP bioluminescence assay kit, per manufacturer’s instructions (Sigma-Aldrich, St. Louis, MO). Final calculations were evaluated using the standard curve, background subtraction and normalization to controls.

### *In vitro* endothelial cell experiments

C57BL/6 murine primary aortic endothelial cells (ECs, Cell Biologics, Catalog No. C57-6052; Chicago, IL) were used for cell culture experiments. ECs were incubated with ectonucleotidase triphosphate diphosphohydrolase-1 (CD39/NTPDase (1) inhibitor, ARL67156 (300 μM; Tocris Bioscience) for 30 min at 37°C. Cells were exposed to transient elastase treatment (0.4 U/ml) for 5 min followed by replacing the media, or treated with cytomix (IL-1β + HMGB1 + IL-17; R&D Systems, Minneapolis, MN; 50 μM), as previously reported (13). Culture supernatants were analyzed for extracellular (e)ATP release, as described above. In separate experiments, transmigration of neutrophils across the endothelium was evaluated using a transmigration assay kit (Cell Biolabs, San Diego, CA). Primary neutrophils (Ly6G +) were isolated from mouse spleens using a Neutrophil Isolation Kit (Miltenyi Biotec, Auburn, CA), and labeled with fluorescent LeukoTracker dye. These labeled neutrophils were then cultured with ECs that were pre-treated with/without cytomix or SPL (50 μM; Sigma Aldrich, St. Louis, MO). Also, in separate experiments, conditioned media transfer (CMT) was performed using ECs and primary F4/80^+^ macrophages (Miltenyi Biotec, Germany) where ECs were exposed to transient elastase treatment with/without apyrase (10 U/ml; Sigma Aldrich) or SPL. After 6 h, CMT was performed to macrophage cultures and supernatants were analyzed after 24 h for IL-1β (R&D Systems) and HMGB1 expressions (IBL International, Hamburg, Germany) by ELISA.

### Cytokine and MMP2 evaluation by multiplex assay

Quantification of cytokine expression and MMP2 activity in murine aortic tissue and culture supernatants was performed using the Luminex Bead Array technique using a multiplex cytokine panel assay (Bio-Rad Laboratories, Hercules, CA), as previously described (13, 30).

### *In vitro* aortic smooth muscle cells experiments

Primary aortic smooth muscle cells (SMCs) were purified from C57BL/6 mice as previously described (31). CMT was performed using ECs and SMCs, where ECs were exposed to transient elastase treatment with/without apyrase (10 U/ml; Sigma Aldrich) or SPL treatments. After 6 h, CMT was performed from ECs to SMCs treated and supernatants were analyzed for cytokine expression and MMP2 activity, as described above.

### Statistical analysis

Statistical evaluation was performed using GraphPad Prism 8 software (GraphPad, La Jolla, CA) and values are presented as the mean ± standard error of the mean (SEM). Parametric one-way ANOVA followed by Tukey’s multiple comparison test was performed to compare differences between three or more groups. A value of *P* < 0.05 was considered statistically significant.

## Results

### Pharmacological inhibition by spironolactone decreases AAA formation

**FIGURE 1:**
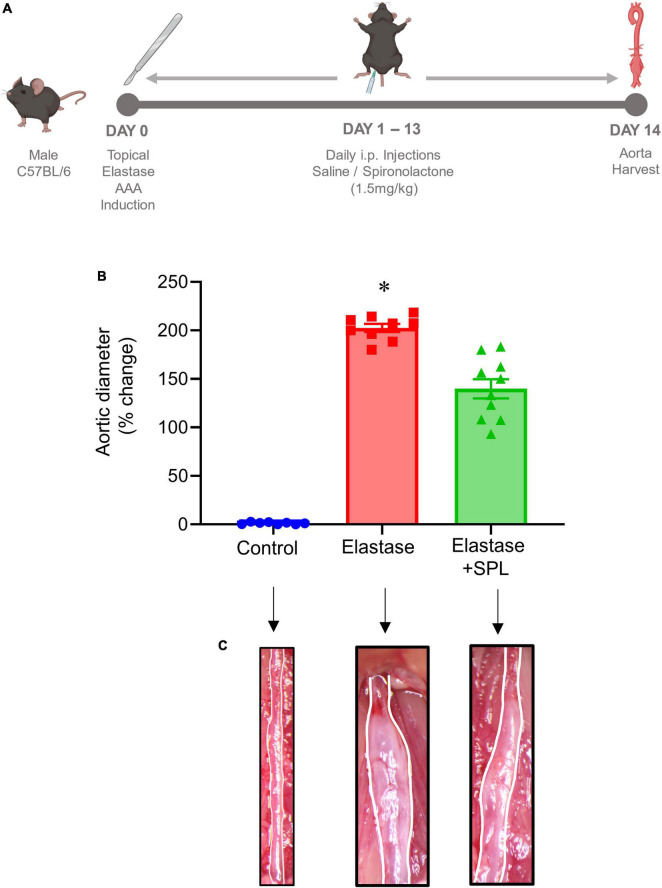
Pharmacological inhibition by spironolactone decreases AAA formation in the topical elastase murine AAA model. **(A)** Schematic depicting the timeline and treatment protocol for spironolactone (SPL) in the topical elastase model of AAA in male C57BL/6 (WT) mice. **(B)** SPL treatment mitigates the increase in aortic diameter observed in elastase-treated WT mice compared to untreated mice. *P < 0.0001 vs. all other groups; *n* = 8–10/group. **(C)** Representation of aortic phenotype in comparative groups.

**FIGURE 2:**
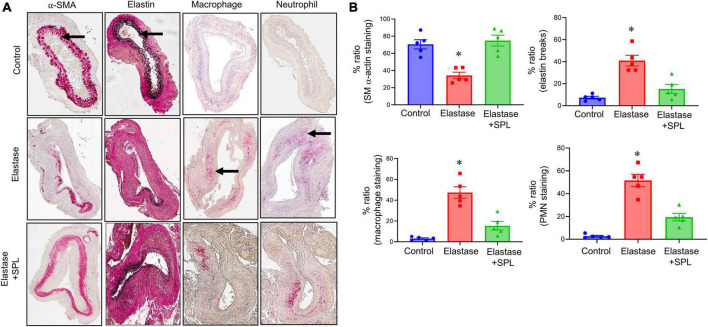
Spironolactone treatment protects the aortic morphology during AAA formation. **(A)** Comparative histology displayed a marked increase in SMα-actin expression, as well as decrease in elastin fiber disruption (VVG staining) and leukocyte (macrophage and PMN) infiltration in SPL-treated mice compared to untreated mice. Arrows indicate areas of immunostaining. *n* = 5/group. **(B)** Quantification of immunohistochemical staining demonstrates a significant increase in SMα-actin expression, decrease in elastin degradation, and leukocyte infiltration in SPL-treated aortic tissue compared to elastase-treated mice alone. *P < 0.01 vs. other groups.

**FIGURE 3:**
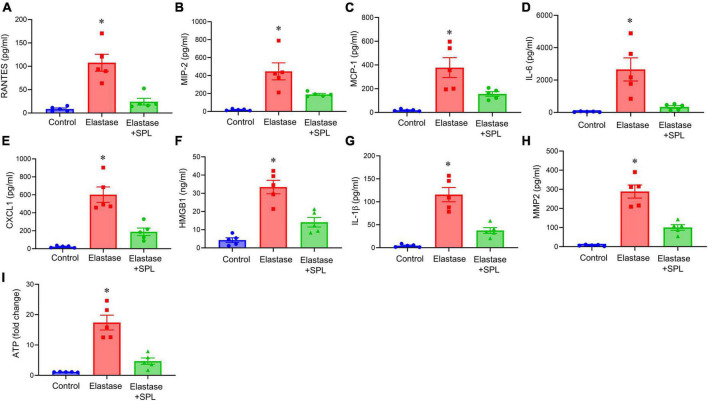
Aortic inflammation during AAA formation is mitigated by spironolactone treatment. **(A–G)** Cytokine expression analyzed in the aortic tissue showed a significant mitigation after SPL administration compared to elastase-treated mice alone on day 14. *P < 0.02 vs. respective controls; *P < 0.05 vs. other groups; *n* = 5/group. **(H)** SPL-treated mice decreased MMP2 activity in aortic tissue compared to elastase-treated mice alone. *P < 0.001 vs. other groups; *n* = 5/group. **(I)** ATP content in aortic tissue was decreased after SPL treatment. *P < 0.002 vs. other groups; *n* = 5 mice/group.

To investigate if treatment using a Panx 1 inhibitor, spironolactone (SPL), can mitigate AAA formation, we used the murine topical elastase treatment model. Aortic diameter was significantly increased in elastase-treated WT mice compared to heat-inactivated elastase (controls) mice (202.5 ± 4.1 vs. 1.3 ± 0.36%; *P* < 0.0001). Treatment with SPL significantly attenuated the aortic diameter in elastase-treated WT mice compared to elastase-treatment alone on day 14 (139.8 ± 9.9% vs. 202.5 ± 4.1%; *P* < 0.0001; Figures 1A–C). No significant dose-dependent differences were observed in aortic diameter after treatment with spironolactone (Supplementary Figure 1). Moreover, a marked increase in SMα-actin expression, decrease in elastin fiber disruption as well as immune cell (macrophage and neutrophil) infiltration was observed in SPL-treated WT mice compared to elastase-treated WT mice alone (Figure 2). Similarly, pro-inflammatory cytokine expression was significantly attenuated in elastase-treated WT mice administered with SPL compared to elastase-treated WT mice alone (Figures 3A–G). Vascular remodeling was evaluated by MMP2 activity which was mitigated in SPL-treated mice compared to elastase-treated mice alone (Figure 3H). Next, we measured the ATP content in aortic tissue, and observed that it was significantly decreased in SPL-treated mice compared to untreated WT mice (Figure 3I).

### Spironolactone treatment decreases aneurysm growth in a chronic AAA model

**FIGURE 4:**
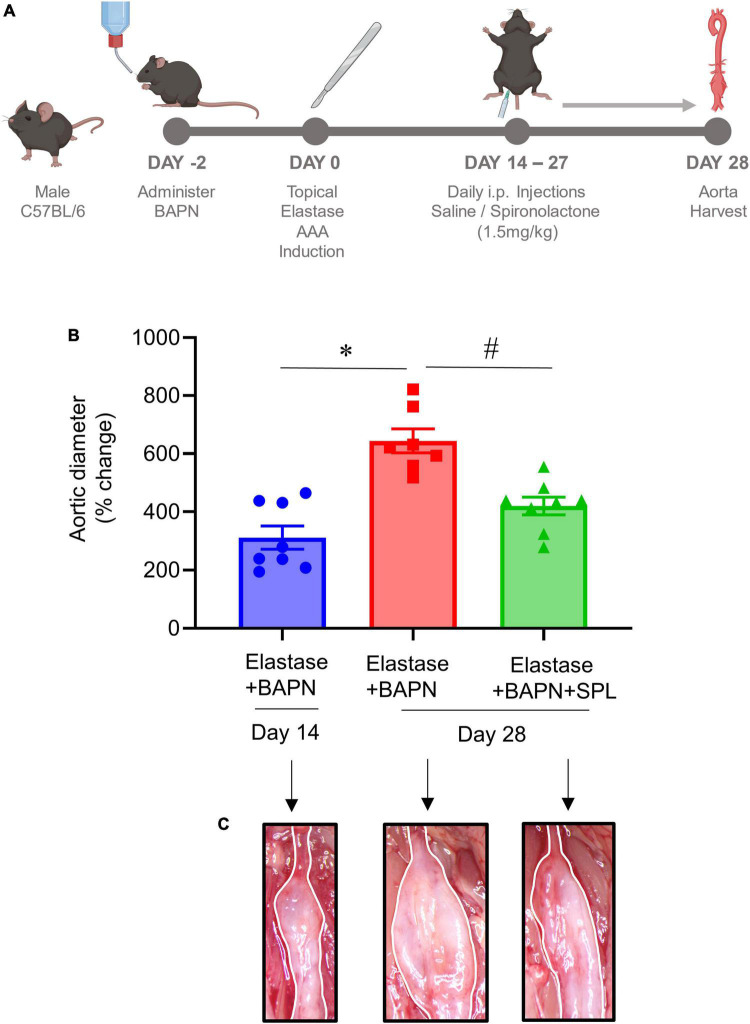
Spironolactone inhibits aortic aneurysms in a chronic AAA model. **(A)** Schematic depicting the chronic AAA and aortic rupture model with elastase + BAPN treatment in WT mice. SPL treatment was administered in mice with pre-formed AAAs from day 14 onwards till harvest. **(B)** SPL treatment decreases aortic diameter of pre-formed aneurysms in elastase + BAPN-treated WT mice compared to elastase + BAPN-treated mice alone on day 28. *P < 0.0001; ^#^*P* = 0.001; *n* = 7–8/group. **(C)** Comparative representation of aortic phenotype in respective groups.

**FIGURE 5:**
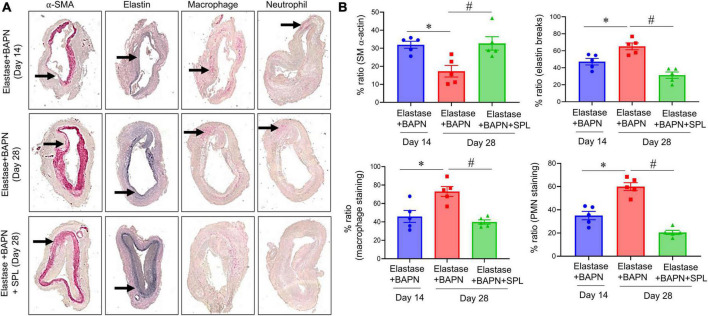
Spironolactone treatment inhibits leukocyte infiltration and preserves the aortic morphology during chronic AAA formation. **(A)** Comparative histology displayed a marked increase in SMα-actin expression, as well as decrease in elastin fiber disruption and leukocyte (macrophage and PMN) infiltration in SPL-treated mice. Arrows indicate areas of immunostaining *n* = 5/group. **(B)** Quantification of histological immunostaining demonstrated a significant preservation of aortic integrity and decrease in immune cell infiltration in SPL-treated aortic tissue compared to untreated mice. *P < 0.02; ^#^*P* < 0.01.

**FIGURE 6:**
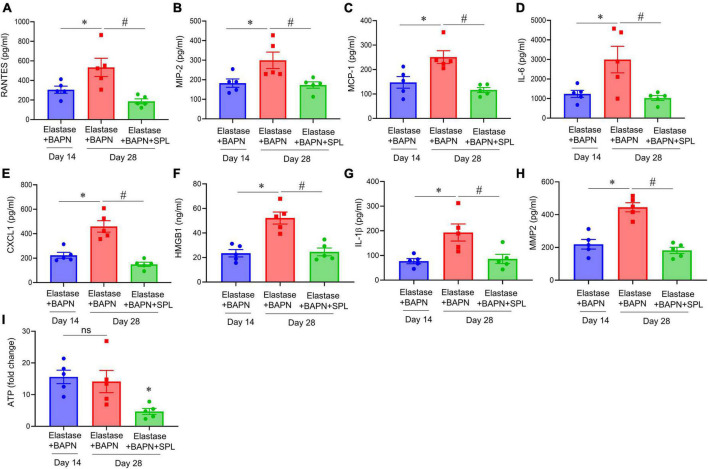
Spironolactone inhibits aortic inflammation and vascular remodeling during chronic AAA formation. **(A–G)** Cytokine expression in aortic tissue is significantly decreased in elastase + BAPN-treated mice after SPL administration compared to untreated mice. *P < 0.04; ^#^*P* < 0.02; *n* = 5/group. **(H)** MMP2 activity was significantly attenuated in SPL-treated mice compared to elastase-treated mice alone. *P < 0.01; ^#^*P* < 0.001; *n* = 5/group. **(I)** ATP content was decreased in SPL-treated mice compared to untreated mice. ns, not significant; *P < 0.01; *n* = 5 mice/group.

We have recently described a chronic, advanced staged AAA murine model with characteristics of persistent aneurysm growth, thrombus formation, and rupture using a combination of peri-aortic elastase and oral BAPN treatment (32). This elastase + BAPN model of chronic AAA was used as a second model to investigate if pharmacological inhibition by SPL can treat “pre-formed” AAAs. WT mice exposed to elastase + BAPN displayed a significant increase in aortic diameter on day 28 compared to day 14 (643.8 ± 41.3 vs. 311.4 ± 40.1%, *P* < 0.0001). Importantly, administration of SPL from days 14 to 27 in mice with existing AAAs significantly decreased the aortic diameter (420.2 ± 30.5 vs. 643.8 ± 41.3%, *P* = 0.001; Figures 4A–C). Similarly, SPL-treated mice demonstrated significant increase in SMα-actin expression, decrease in elastic fiber disruption as well as less infiltration of macrophages and neutrophils, compared to untreated mice on day 28 (Figure 5). A significant attenuation of pro-inflammatory cytokines, including IL-1β and HMGB1, associated with aneurysm formation were seen in the SPL-treated mice compared to saline-treated mice (Figures 6A–G). Moreover, SPL-treated mice showed decrease in MMP2 activity and aortic tissue content of ATP compared to untreated controls (Figures 6H, I). Taken together, this data shows that spironolactone administration decreases the growth of pre-formed aneurysms by inhibiting aortic inflammation, MMP activity and ATP content of aortic tissue.

### Spironolactone inhibits endothelial cell-dependent ATP release to decrease neutrophil transmigration

**FIGURE 7:**
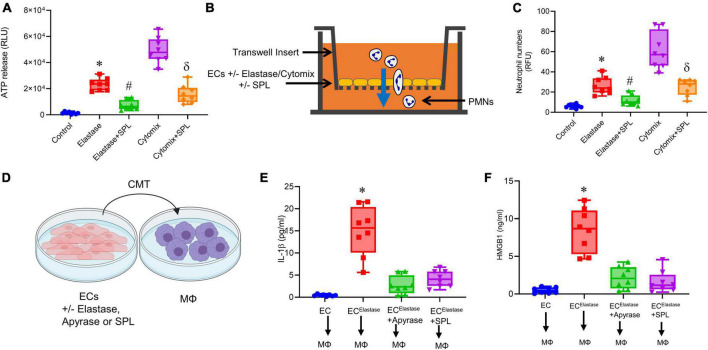
Spironolactone decreases EC-mediated eATP release to mitigate macrophage activation. **(A)** eATP release by ECs after transient elastase-exposure or cytomix-treatment is attenuated by SPL compared to untreated controls. *P < 0.0001 vs. control; ^#^*P* < 0.0001 vs. elastase; ^δ^*P* < 0.001 vs. cytomix; *n* = 8/group. **(B)** Schematic of fluorescent-labeled polymorphonuclear neutrophils (PMNs) trafficking through ECs using a transwell assay. **(C)** SPL treatment of ECs significantly decreased neutrophil transmigration after transient elastase- or cytomix-treatment. *P = 0.002 vs. control; ^#^*P* = 0.04 vs. elastase; ^δ^*P* < 0.0001 vs. cytomix; *n* = 8/group. **(D)** Conditioned media transfer (CMT) from elastase-treated ECs to macrophages with/without SPL or apyrase pre-treatment. **(E,F)** SPL treatment of ECs inhibited upregulation of IL-1β and HMGB1 secretion by macrophages after CMT from ECs. *P < 0.0001 vs. all other groups; *n* = 8/group.

To evaluate the effect of SPL treatment on ECs, transient elastase treatment of ECs was performed and eATP was measured after 6 h. ECs were also treated with cytomix (relevant pro-inflammatory cytokines in AAA pathogenesis, IL-1β + IL-17 + HMGB1), as previously described (13, 26, 27, 30). A multifold upregulation in eATP release was observed by elastase- or cytomix-treated ECs compared to controls at 6 h that was decreased by SPL treatment (Figure 7A). In separate cultures, neutrophil trafficking was analyzed in cultured ECs. Elastase or cytomix-treatment significantly increased neutrophil transmigration, which was mitigated by SPL treatment (Figures 7B, C).

### Spironolactone treated ECs inhibits macrophage activation

Using *in vitro* experiments, the communication between ECs and macrophages was analyzed by conditioned media transfer (CMT) (Figure 7D). CMT from elastase-treated ECs to macrophages resulted in significant increase in IL-1β (14.9 ± 1.9 vs. 0.4 ± 0.07 pg/ml; *p* < 0.0001; Figure 7E) and HMGB1 (8.4 ± 1.0 vs. 0.48 ± 0.1 ng/ml; *p* < 0.001; Figure 7F) expression compared to controls, respectively. CMT from ECs after pre-treatment with apyrase (10 U/ml), or SPL (50 μM) resulted in attenuation of IL-β (2.7 ± 0.7 and 4.1 ± 0.6 pg/ml; *p* < 0.0001) and HMGB1 (2.1 ± 0.5 and 1.6 ± 0.5 ng/ml, respectively) release from macrophages (Figures 7E, F). This data shows that spironolactone mitigates EC-macrophage crosstalk by inhibiting eATP release from ECs to decrease macrophage activation.

### Spironolactone treatment mitigates SMC-dependent activation and remodeling

**FIGURE 8:**
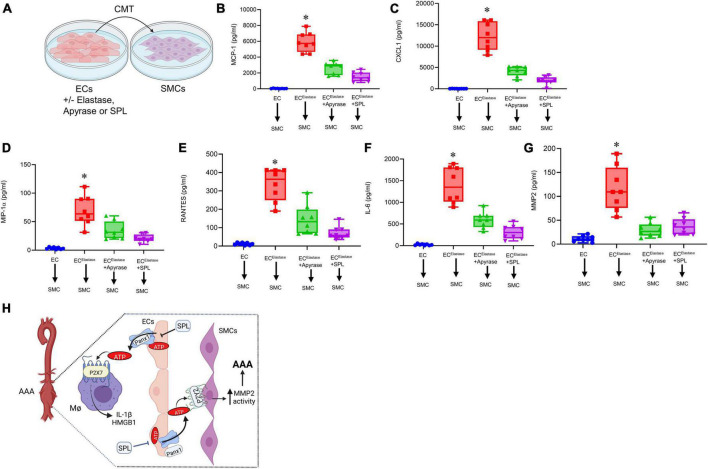
Spironolactone treatment inhibits SMC activation *via* decreased eATP release from ECs. **(A)** Crosstalk of ECs with SMCs was evaluated by CMT from elastase-treated ECs to SMCs with/without pre-treatment with SPL. **(B–F)** Cytokine secretion was upregulated by SMCs after CMT from ECs, that was attenuated by pre-treatment of ECs with apyrase or SPL. *P < 0.0001 vs. all other groups; *n* = 8/group. **(G)** MMP2 activity by SMCs was significantly attenuated by pre-treatment of ECs with SPL. *P < 0.0001 vs. other groups; n = 8/group. **(H)** Hypothetical schematic describing the molecular signaling events during SPL-mediated protection against AAA formation. SPL treatment attenuates EC-mediated eATP release from Panx 1 channels to decrease purinergic signaling on macrophage-dependent IL-1β and HMGB1 secretion. Also, SPL treatment mitigates eATP release from ECs to downregulate SMC-dependent pro-inflammatory cytokine secretion and MMP2 activity. Collectively, SPL-treatment attenuates vascular inflammation and remodeling to inhibit the progression of AAA formation. ECs, endothelial cells; SMCs, smooth muscle cells; HMGB1, high mobility group box 1; MΦ, macrophages; SPL, spironolactone.

The crosstalk of ECs with SMCs *via* Panx 1/ATP signaling can lead to activation and remodeling of SMCs. To investigate the protective role of spironolactone in modulating the EC-SMC crosstalk, CMT was performed from elastase-treated ECs to SMCs in a cell culture model (Figure 8A). Elastase-treated ECs induced a significant increase in pro-inflammatory cytokine secretion as well as MMP2 activity from SMCs, which was mitigated by pre-treatment of ECs with apyrase or SPL (Figures 8B–G). Taken together, these results demonstrate that spironolactone treatment can inhibit macrophage activation and SMC-dependent remodeling *via* blockade of eATP release by ECs (Figure 8H).

## Discussion

This study elucidates the potential of spironolactone as a therapeutic option for the attenuation of growth and progression during AAA formation. Our results indicate that spironolactone has the ability to decrease EC-mediated eATP release and decrease the subsequent crosstalk with macrophages as well as SMCs to alter the progression of AAAs. Importantly, pharmacological treatment with a spironolactone significantly inhibits inflammation and remodeling of the aortic tissue to attenuate aneurysm formation in two separate established models of experimental murine AAA. *In vivo* experiments exhibited that spironolactone can decrease ATP content, macrophage-specific cytokine secretion and SMC-specific MMP2 activity in aortic tissue of WT mice. Furthermore, *in vitro* experiments demonstrated that spironolactone can disrupt the crosstalk of ECs with macrophages and SMCs to downregulate key inflammatory cytokines, ATP release and MMP2 activity. Collectively, these results suggest that treatment with spironolactone can target EC-mediated ATP release to inhibit aortic inflammation and remodeling to decrease the progression of AAAs and prevent impending aortic ruptures.

The hallmarks of AAAs is characterized by increased pro-inflammatory cytokine milieu, leukocyte transmigration and activation, enhanced matrix metalloproteinase activity, elastin degradation and loss of smooth muscle cell α-actin integrity (26, 29, 30). The complexities of these signaling events culminates in disrupted integrity of aortic vascular wall and dilation of the vessel, that can eventually lead to aortic rupture and sudden death. The progression to aortic rupture in this chronic pathology depends on the rate of aneurysm expansion, thereby providing an opportunity of pharmacological intervention to immunomodulate the progression of AAAs. Our recent studies have elucidated the previously unrecognized role of ion channels such as Panx 1 and transient receptor potential vanilloid 4 (TRPV4) in the pathogenesis of AAA formation (13, 33). Pharmacologic inhibition of these ion channels could potentially be harnessed for slowing AAA progression thus sparing patients from requiring surgical interventions (13, 33). We recently described that EC-dependent Panx 1 channels play a pivotal role in immune cell activation and SMC-dependent remodeling during AAA formation (13). One of the key soluble paracrine factors released by ECs *via* Panx 1 channels is eATP that has the ability to initiate purinergic signaling on relevant immune cells triggering a cascade of pro-inflammatory signaling events that culminates in vascular remodeling (34–36).

The release of eATP from EC-Panx 1 channels has been described to illicit a pivotal role as a danger associated molecular pattern (DAMP) molecule that triggers inflammatory signaling in various pathological conditions (13, 37, 38). eATP can activate P2 purinergic receptors (P2Rs) and has been shown to be degraded by ecto-nucleotidases to generate adenosine, that can act as a potent immunosuppressant to dampen inflammation (39). Panx 1-mediated eATP release can activate immune cells *via* purinergic P2X and P2Y receptors to upregulate tissue inflammation (25, 40). Tissue inflammation during AAA involves a pivotal role of cytokines that are macrophage-dependent, such as inflammasome (IL-1β) and high mobility group box 1 (HMGB1) secretion (26, 27, 41). It is plausible that other cells such as fibroblasts may exert functional changes on intimal and medial cell populations, i.e., ECs, macrophages, and SMCs. However, our study focuses on the Panx 1 channel activation on ECs, based on our recently published study, that could in turn may result in fibroblast activation (13). In this study, we demonstrate that inhibition of eATP release, likely from Panx 1 channels, by spironolactone downregulates the intercellular communication leading to significantly reduced macrophage and SMC activation.

Recent reports suggest that Panx 1-mediated ATP release regulates vascular function in arterial vasculature (16, 42). Purines such as ATP are known to trigger extracellular signaling by acting as danger-associated molecular pattern molecules and mediate tissue inflammation. Therefore, blockade of ATP release by Panx 1 channels signifies an important pharmacological tool that could be harnessed to mitigate vascular pathologies. Recently, several pharmacological compounds, such as spironolactone, have been suggested to exhibit selectivity in inhibiting Panx 1 currents (23). Spironolactone is an effective anti-hypertensive compound that can significantly inhibit both human and mouse Panx 1 channels. Although the anti-hypertensive actions of spironolactone are postulated to inhibit α1-adrenergic receptor-mediated vasoconstriction of resistance arteries, it is plausible that Panx 1 inhibition may be associated with decrease in aneurysm formation due to reduction in mean arterial pressure. Panx 1 regulation in conduit arteries has been suggested to modulate tone of vasculature which could be the mechanistic pathway through which spironolactone modulates aortic inflammation and remodeling during AAAs (43). This is supported by our clinical data observation which demonstrated a correlation between Panx 1 inhibitors, such as spironolactone, and a significant decrease in overall mortality in AAA patients (13). Additionally, spironolactone is a non-selective mineralocorticoid receptor antagonist that can bind to the progesterone, androgen, and mineralocorticoid receptors (44). Previous studies have shown the beneficial effects of mineralocorticoid receptor antagonists such as eplerenone on suppression of aortic aneurysm progression through an anti-inflammatory effect (45). Our present study supports and provides evidence for spironolactone to effectively attenuate aortic inflammation and vascular remodeling, thereby suggesting the potential of clinical translation in AAA patients to reverse the progression of existing AAAs and mitigate the risk of aortic ruptures.

The limitations of experimental murine models involve lack of chronicity observed in the clinical scenario for AAAs. Although the topical elastase model replicates the main hallmarks of aortic inflammation observed in AAAs, it lacks chronic inflammation, thrombus formation, and aortic rupture seen in human disease progression. Therefore, our study utilized a second chronic model of AAA involving elastase + BAPN treatment that involves progression of AAAs to aortic rupture which supported our hypothesis and enhances the clinical translation of this study (32). Further studies with pre-clinical porcine models are required to enable the delineation of cellular mechanisms of the disease process in AAAs, and characterize the long term safety and efficacy of the dose regimen required for large mammals, which is not feasible in the murine models. Another advantage of our recently described swine AAA model is that it exhibits the sequelae of chronic aneurysmal disease such as thrombus formation and rupture (46). The clinical feasibility of compounds like spironolactone should be delineated in the pre-clinical, large animal models to delineate relevant strategies for human studies.

In summary, this study demonstrates that spironolactone can be effectively used for the significant mitigation of vascular inflammation and remodeling to inhibit the AAA progression. The cellular mechanism of spironolactone-mediated protection involves decreased eATP release from ECs that downregulates macrophage activation and smooth muscle cell-remodeling to protect against progression of AAAs. Further studies in our recently characterized large animal model will delineate the feasibility of spironolactone for clinical translation of this experimental therapeutic strategy in vascular pathology of aneurysms and aortic rupture.

## Data availability statement

The raw data supporting the conclusions of this article will be made available by the authors, without undue reservation.

## Ethics statement

This animal study was reviewed and approved by University of Florida’s Institutional Animal Care and Use Committee (protocol #202110051).

## Author contributions

ZL, GS, JH, GL, SH, and AS conducted experiments and acquired data. GU and AS edited the manuscript, supervised experiments, analyzed and interpreted the data, drafted the manuscript, and designed the study. All authors critically reviewed and edited the final version of the manuscript.
