# Supplementary material for: Pharmacologic inhibition by spironolactone attenuates experimental abdominal aortic aneurysms
Source: Front Cardiovasc Med. 2023 Jan 26;10:1101389. doi: 10.3389/fcvm.2023.1101389 (PMC9908993; doi:10.3389/fcvm.2023.1101389)
Supplement: Supplementary file 1 [file Image_1.pdf]

Supplementary Material

Pharmacologic inhibition by spironolactone attenuates experimental abdominal aortic aneurysms

Zachary Ladd, Gang Su, Joseph Hartman, Guanyi Lu, Sara Henseley, Gilbert R. Upchurch, Jr., & Ashish K. Sharma

Table of Contents:

Supplementary Figure S1

Supplementary Figure S1

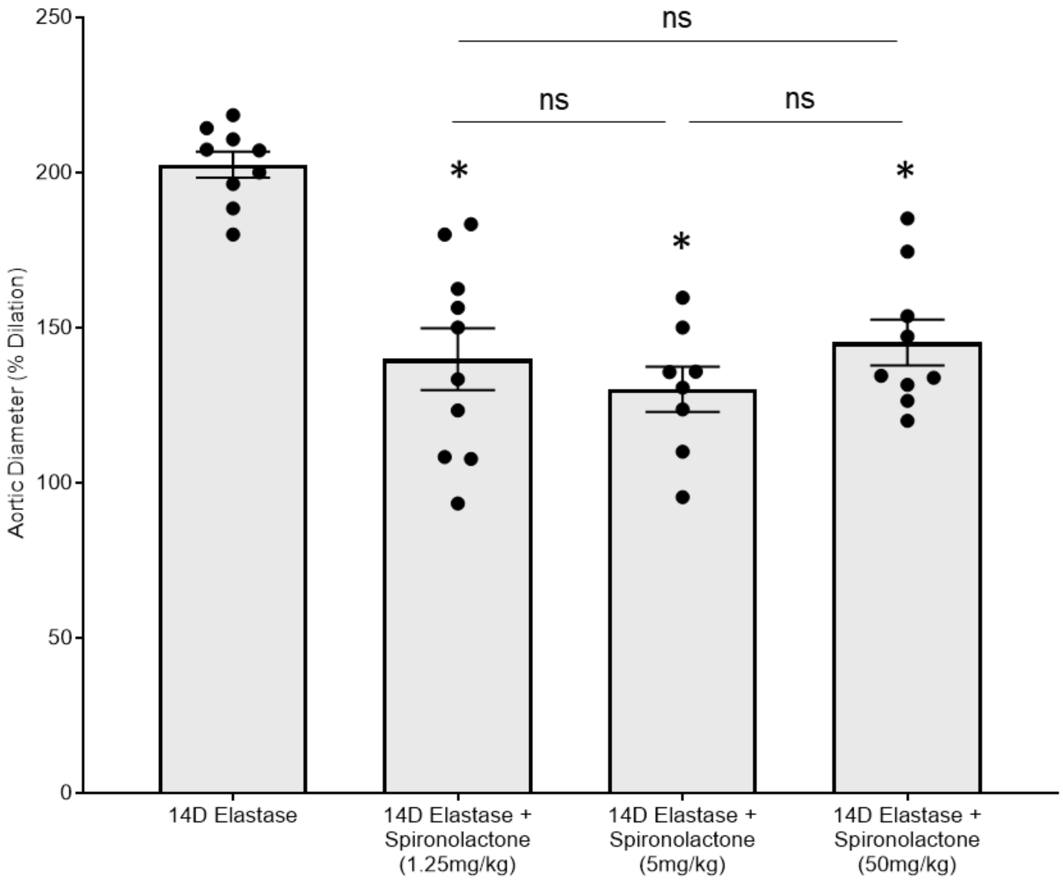

Supplementary Fig. S1. Treatment with spironolactone mitigates the increase in aortic diameter observed in elastase-treated WT mice compared to untreated mice on day 14. However, there was no dose-dependent change with spironolactone treatment on the aortic diameter after elastase-treatment compared to untreated controls. **P*<0.0001 vs. elastase; ns, not significant; n=8-10/group.
